# Sustainable communities: we are the world

**DOI:** 10.1038/s41598-025-91952-7

**Published:** 2025-03-08

**Authors:** Idiano D’Adamo

**Affiliations:** Department of Computer, Control and Management Engineering, Sapienza University of Rome, Rome, Italy

**Keywords:** Sustainability, Socioeconomic scenarios

Sustainable communities represent a social model that transcends individualism, prioritising a collective mission that balances respect for older generations with trust in the transformative potential of younger ones. At its core, sustainability is a collaborative, altruistic philosophy, embodying the essence of a ‘we’-oriented approach to life. It is a guiding principle, akin to the morning star, illuminating the path forward. When young people are positioned as agents of change, their voices and perspectives shape pragmatic, forward-looking solutions. These solutions emphasise a green and circular vision, fostering energy and material independence to achieve autonomy, enhance competitiveness and mitigate geopolitical risks. In this context, skills development is a critical enabler, empowering communities to overcome inertia, cultivate teamwork and balance local commitments with a global perspective.

This editorial reflects on a crucial question: Will humanity learn from its past mistakes? The discussion aims to provide insight into the concept of sustainability and the strategic significance of the United Nations’ Sustainable Development Goals (SDGs), which must be central to government agendas and closely monitored over time.

The concept of sustainable development, as first articulated in the 1987 Brundtland Report, does not stop at environmental protection alone, but emphasises a holistic balance among environmental stewardship, economic opportunity and social well-being. In 2000, San Giovanni Paolo II’s spiritual testament called for morning sentinels to serve as vigilant stewards of life, safeguarding it at every stage to ensure a habitable future for all. Similarly, in 2015, the European Environment Agency highlighted that the circular economy encompasses waste management, waste prevention and resource efficiency, while the green economy also integrates human well-being and ecosystem resilience. In the same year, Papa Francesco’s encyclical *Laudato si* highlighted the interconnectedness of environmental and social degradation, emphasising their disproportionate impact on the world’s most vulnerable populations.

This editorial invites citizens to reflect deeply on two fundamental questions:

1. How many of us are genuinely willing to devote our time to listening to and addressing the concerns of those seeking human support?
2. Are we prepared to make sacrifices to ensure that contemporary youth have access to meaningful opportunities in the present?

The green transition represents a pragmatic pathway to sustainability, and one that must be undertaken in collaboration with younger generations. However, the prevalence of greenwashing among governments, corporations and even individuals undermines genuine progress. In the digital age, there is a troubling tendency to prioritise superficial gestures—such as garnering social media likes—over meaningful, concrete actions. Thus, there is a growing need to move beyond an ideological perspective to embrace a problem-solving approach that presents sustainability not as the enemy of progress, but as its enabler. Key pillars of this transformation include stakeholder engagement, behavioural nudges and sustainable education to foster a shift in societal values and communication. In this context, it is not only the circulation of resources that is needed, but also innovative ideas that challenge narratives promoting war or totalitarian behaviour.

The ecological transition demands the active participation of young people and the full utilisation of talent across all sectors. The ‘green premium’ reflects the additional value consumers attribute to products or services derived from renewable sources rather than fossil fuels. Similarly, the ‘circular premium’ signifies the value generated and recognised through the use of reused, recycled and recovered materials in place of polluting alternatives.

## Article highlights in the collection

Climate change, natural disasters and human self-interest have compelled urban and rural communities to rethink their approaches to resource management. Sustainable communities aim to achieve energy independence, balance their use of natural resources and preserve both cultural and natural heritage, all while maintaining productivity. These efforts support economic resilience and empower citizens to actively participate in the green transition. This collection features multidisciplinary research contributions aligned with SDGs 7, 8, 9, 11 and 12, comprising 18 articles that explore diverse perspectives on sustainability.

University alliances may support the development of sustainable local communities by fostering the training of future professionals with a strong commitment to sustainability^1^. Such training may integrate socially responsible entrepreneurship with a long-term perspective, aimed at strengthening communities^2^. Sustainable communities within academic settings are based on six pillars: sustainable education, energy (and resource) independence, subsidies to support the green economy, initiatives to reduce the carbon footprint, the development of energy communities and the creation of new green professional opportunities^3^.

Cities are facing increasing pressure to address social and ecological transitions by promoting collaborative approaches among diverse stakeholders. Such approaches aim to foster accessibility to urban natural spaces and create opportunities for residents to engage with such landscapes^4^. Urban planners are tasked with developing solutions to reduce cities’ carbon footprints through the joint use of renewable energy and energy efficiency measures^5^. Additionally, sustainable urban transportation systems must prioritise efficiency, with green choices significantly dependent on the relative costs of fossil fuel^6^. In rural communities, resilience can be enhanced through the efficient transmission of information and the adoption of prevention practices^7^. In addition, the benefits associated with sustainable business models and their transformative impacts on value chains should be demonstrated^8^. However, the correct balance of urban–rural development requires further investigation, and carbon market data must be integrated into the decision-making process^9^.

Energy communities may play a pivotal role in advancing low-carbon economies. However, this transition cannot rely on technological advancements alone; it also requires policies designed to overcome social barriers to change^10^. While small-scale renewable energy systems may present scalability challenges in densely populated regions, the integration of technological innovation, novel market mechanisms and active community participation offers promising solutions^11^. Social capital analysis provides valuable insights into the evolution of renewable energy communities, revealing that civic norms within households, social trust and structural interactions within neighbourhoods constitute the social contexts in which these communities develop and thrive^12^.

Another critical strategic concept is the circular economy, which serves as an indicator of a state’s commitment to fostering sustainable communities^13^. In this direction, the development of effective methods and approaches is essential for identifying reference targets and conducting analyses aimed at achieving eco-efficiency goals^14^.

However, research underscores the relevance of assessing social attitudes, highlighting the need for an inclusive and personalised approach to support the growth of sustainable communities^15^. Decision-makers’ initiatives, incentives and strategies must be evaluated in relation to the specific nature of the populations to which they cater, including their personal and social characteristics^16^. Moreover, robust models are needed to integrate diverse data points, such as environmental resources, economic indicators (e.g., housing prices)^17^ and the interplay between environmental uncertainty and digital technologies^18^.

**Fig. 1:**
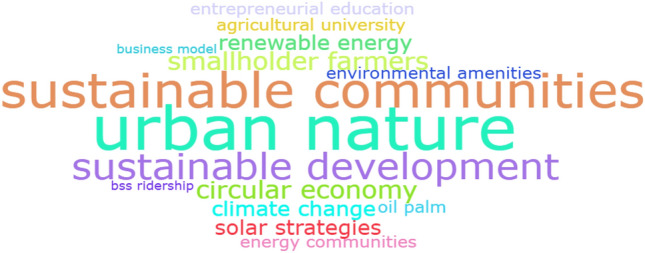
Word cloud—‘Sustainable communities’ collection obtained using R-tool.

Figure 1 presents a word cloud generated from the abstracts of all the papers in this collection, illustrating the prominence of three recurring concepts: (i) sustainable communities, (ii) sustainable development and (iii) urban nature.
